# Editorial

**Published:** 1916-04

**Authors:** 


### EDITORIAL

The Atlanta Journal-Record of Medicine has its Business Office at 23 West Alabama Street where all business communications should be sent.

Remittances are payable to The Journal-Record of Medicine.

Concerning Original Communications and Editorial matters, address Richard R. Daly, M. D., 708 Empire Life Building, Atlanta, Ga.

Reprints of Original Articles will be furnished at cost price. Requests for them had best be made on the Manuscript.

Upon request, we will present, postpaid, to each contributor of an original article, twenty copies of The Journal-Record of Medicine containing such article.

---

### GOOD FAITH.

These surely are the times that try men's souls. Human nature shows its instability in conventional development and seems all over the world to be throwing off the restraints that communal and gregarious life have evolved. War and betrayals, revolutions and rebellions have made the program and are arising on nearly every hand during the last two years. The glut of production had so long set aside and denied the glee of contest that when the fight did come it could not be satisfied by anything but a lust of blood and a destruction of all that had led or seemed to lead to moral and social control. Common honesty disappeared at the approach of the acid test and left nothing fast but the primitive stuff that destroys all else but itself and then dies in proliferation. Whole nations were at war and striving for their own isolation. Each wanted to be master and make the others keep aside. Primitive men have to run wild at times even after the tribes have been formed, and modern man is so close to them that everyone understands what it means to want to be alone. To be alone is to be master and to repel or conquer those who approach is to continue master. It is the spirit within us that the generations of culture have not outlived.

We have learned that by communal existence and the giving up of personal things for the benefit of the general, we are happier and better. We have partly learned to balance selfishness and unselfishness and we have called it civilization. Since all the results had to do with the future of either the next hour of the next year, there had to be some bond between men and they set up and used good faith. It worked. As it grew in strength it worked better and nations resulted. In the nations and among the people, commerce spread because of its agency and science found room for itself. Men could see into the future with a fair degree of confidence and they depended upon each other for helpfulness in study and investigation having the security of honor, the child of good faith, that their work would be their own and that their efforts would not be forfeited to trespassers.

In the art and science of medicine more perhaps than anywhere else the element of good faith contibuted to growth and development. The students of life's great problems have felt the immensity of their tasks and have always been most eager to tell all they knew to their fellows in the growing hope that mutual learning would lead all to a light all needed and all could rejoice in. The advancement in medicine since this Journal has been in existence has been more marvellous than its early editors could have imagined and even in that brief space of time good faith has been proven by its works.

Trials have come and been withstood. The profession today seems stronger and better than ever because of its elimination of cant and hypocrisy and of the setting aside of traditions based upon unproved empiricism. It is simplifying itself and apparently is ready always to look itself in the face and to do constructive criticism whenever the opportunity comes.

But are we secure? When the spirit of war is rampant all around us and men are betraying their brothers and themselves in riotous rebellions as in Ireland and in politics in this country, are we enough removed by the all absorbing nature of our work, dealing as it does with every phase of human happiness and woe and even with life and death itself, are we distant enough from the primal human influences to be safe?

The country is on the verge of war. Our troops are in Mexico in a delicate situation where only consummate skill can take them without war with a high strung neighbor. The President is already striving for peace while he insists upon our rights and upon the maintenance of our honor even when our hospitality has been betrayed. These are times that try mens' souls, for our patriotism is unbounded and our fighting blood runs fiercely when there is cause. We are not non-resistants by any means. We will struggle and fight with the best of them when the time comes, but shall we not through it all, keep the good faith?

There could be no greater disaster to the world than a loss of good faith among the members of our profession such as we daily see among the members of another group as a concomitant of the war. Our heritage is greater and better than theirs and its benefits are for all. No matter what the reversion, or the struggle or the blood lust of fighting may bring to others, nor how close it may come to ourselves, our work must be done in good faith with each other and not only that but an example set and an influence established that will come to the home of every man when the fighting is over.

